# Recent Advances in Biofilmology and Antibiofilm Measures

**DOI:** 10.1155/2017/5409325

**Published:** 2017-03-15

**Authors:** Nithyanand Paramasivam, Shunmugiah Karutha Pandian, Ariel Kushmaro, Supayang Voravuthikunchai, Aruni Wilson

**Affiliations:** ^1^Biofilm Biology Laboratory, Anusandhan Kendra II, School of Chemical and Biotechnology, SASTRA University, Tirumalaisamudram, Thanjavur, Tamil Nadu 613 401, India; ^2^Department of Biotechnology, Alagappa University, Science Campus, Karaikudi, Tamil Nadu 630 003, India; ^3^Department of Biotechnology Engineering, Ben-Gurion University of the Negev, 84105 Beer-Sheva, Israel; ^4^Department of Microbiology, Faculty of Science, Prince of Songkla University, Hat-Yai, Songkhla 90112, Thailand; ^5^Division of Microbiology and Molecular Genetics, School of Medicine, Loma Linda University, Loma Linda, CA, USA

Biofilms have now been recognized as a predominant life style of several bacteria and fungi, wherein single-cell organisms assume a temporary multicellular lifestyle. A hallmark of biofilms is the formation of an extracellular matrix or EPS (extracellular polymeric substances) that forms a thick layer encasing the microbial cells, thereby protecting them from antimicrobials and from the host immune response. Hence, the treatment of biofilm infections has become a challenge and has attracted significant scientific attention. To address this important issue we therefore present a number of reviews and research papers focusing on the control of biofilms in a special issue of this journal.

One of the papers in the special issue points out that siderophore molecules such as transferrin have a major impact on* Bacillus thuringiensis* biofilms. This is important, as it is well known that members of the genus* Bacillus* survive even in adverse conditions. The study showed for the first time that the molecule transferrin helps* B. thuringiensis* to be established and sustained as biofilms and provides important information regarding the mechanism of biofilm establishment of this bacterium. Another paper reports on the development of a novel antibiofilm dressing technology using carboxymethylcellulose silver-containing dressing and tests its efficacy on several biofilm models. Results showed that this wound dressing was also more effective than the available standard silver dressings in reducing the EPS layer, the protective biofilm component.

Another paper provides a detailed review about the recently developed nanotechnology-based biomaterials to prevent biofilms and discusses various strategies used to make antibiofilm surfaces. The review pointed out that some interesting compounds such as dendrimers have antibiofilm activity and concludes that preparation of cost-efficient nanobiomaterials is the “need of the hour.” An additional review article in this special issue deals with oral biofilm models. Since bacterial species present in the oral cavity predominantly live in a biofilm life style, oral biofilm models have become important to study as to how these bacteria form biofilms or to understand the functioning of oral microcosm in a biofilm. The review gives a detailed account on the pros and cons of the currently existing oral biofilm models and finally advocates that the right model should be chosen based on the rationale to be addressed.

S. Gowrishankar et al. report a very interesting observation about the presence of biofilm forming methicillin-resistant* Staphylococcus aureus* (MRSA) strains from pharyngitis patients. The coexistence of MRSA along with Group A Streptococcus (GAS), the causative agent of pharyngitis, stresses the need for a broad-spectrum antibiofilm agent that acts on both the biofilm inhabiting species. Since biofilms formed by food borne pathogens or food spoilage bacteria on food processing equipment are the main cause for food contamination, two papers address this burgeoning problem in this issue. Till date, it is thought that disinfection of food processing plants by sanitizers is the most feasible option for food industries. L. Cincarova et al. show that usages of sublethal concentrations of disinfectants are not effective in removing biofilms of* S. aureus* isolated from meat processing plants. This study emphasizes the need for optimizing the exact dosage and duration of disinfectants in food industries. An additional common problem encountered in food industries is biofilm formation by* Asaia* sp. on production lines of soft drink plants which eventually contaminates soft drinks even in the presence of preservatives. H. Antolak et al. show that that polyphenolics present in bilberry and blackcurrant juices prevented the adhesive property of* Asaia* sp. The authors further state that bilberry and blackcurrant juices which can prove to be interesting alternatives to artificial additives to keep the microbial stability of final products. Drug resistance by biofilm forming pathogens has reached alarming proportions worldwide. This necessitates the need of new antimicrobial agents, either synthetic or natural products, to treat these recalcitrant infections. The final paper of this issue addresses this important problem wherein B. Fu et al. show that a Chinese medical herb* Herba Patriniae* inhibited the mature biofilms of* Pseudomonas aeruginosa* and also decreased the exopolysaccharide (EPS) production.

*Nithyanand Paramasivam* *Shunmugiah Karutha Pandian* *Ariel Kushmaro* *Supayang Voravuthikunchai* *Aruni Wilson*

